# Adherence to *a priori*-Defined Diet Quality Indices Throughout the Early Disease Course Is Associated With Survival in Head and Neck Cancer Survivors: An Application Involving Marginal Structural Models

**DOI:** 10.3389/fnut.2022.791141

**Published:** 2022-04-25

**Authors:** Christian A. Maino Vieytes, Sandra L. Rodriguez-Zas, Zeynep Madak-Erdogan, Rebecca L. Smith, Katie R. Zarins, Gregory T. Wolf, Laura S. Rozek, Alison M. Mondul, Anna E. Arthur

**Affiliations:** ^1^Division of Nutritional Sciences, University of Illinois at Urbana-Champaign, Urbana, IL, United States; ^2^Department of Animal Sciences, University of Illinois at Urbana-Champaign, Urbana, IL, United States; ^3^Cancer Center at Illinois, University of Illinois at Urbana-Champaign, Urbana, IL, United States; ^4^Department of Food Science and Human Nutrition, University of Illinois at Urbana-Champaign, Urbana, IL, United States; ^5^Department of Pathobiology, University of Illinois at Urbana-Champaign, Urbana, IL, United States; ^6^Department of Environmental Health Sciences, University of Michigan, Ann Arbor, MI, United States; ^7^Department of Otolaryngology, University of Michigan, Ann Arbor, MI, United States; ^8^Department of Epidemiology, University of Michigan, Ann Arbor, MI, United States; ^9^Department of Dietetics and Nutrition, University of Kansas Medical Center, Kansas City, KS, United States

**Keywords:** nutritional epidemiology, marginal structural model, cancer, diet quality, survivorship

## Abstract

No studies, to date, have scrutinized the role of *a priori* dietary patterns on prognosis following a head and neck squamous cell carcinoma (HNSCC) diagnosis. The purpose of this analysis was to evaluate the associations between adherence to six *a priori* defined diet quality indices (including AHEI-2010, aMED, DASH, and three low-carbohydrate indices) throughout the first 3 years of observation and all-cause and cancer-specific mortalities in 468 newly diagnosed HNSCC patients from the University of Michigan Head and Neck Specialized Program of Research Excellence (UM-SPORE). The dietary intake data were measured using a food frequency questionnaire administered at three annual time points commencing at study entry. Deaths and their causes were documented throughout the study using various data sources. Marginal structural Cox proportional hazards models were used to evaluate the role of diet quality, as a time-varying covariate, on mortality. There were 93 deaths from all causes and 74 cancer-related deaths adjudicated throughout the observation period. There was a strong inverse association between adherence to the AHEI-2010, all-cause mortality (HR_*Q5*–*Q1*_:0.07, 95% CI:0.01–0.43, *p*_trend_:0.04), and cancer-specific mortality (HR_*Q5*–*Q1*_:0.15, 95% CI:0.02–1.07, *p*_trend_:0.04). Other more modest associations were noted for the low-carbohydrate indices. In sum, higher adherence to the AHEI-2010 and a plant-based low-carbohydrate index throughout the first 3 years since diagnosis may bolster survival and prognosis in newly diagnosed patients with HNSCC.

## Introduction

Head and neck squamous cell carcinoma (HNSCC) is a class of malignancies primarily implicating the anatomy of the oral cavity, pharynx, and larynx. Collectively, these malignancies are leading forms of cancer characterized by increasing incidence rates in the United States and harbor a low 5-year survival rate of less than 65% (1). The primary etiologic agents include smoking, oral tobacco use, drinking, and infection with carcinogenic strains of human papillomavirus (HPV) although most HPV-positive HNSCCs are driven by HPV-16 (2, 3). In addition, the distribution of HNSCC across the sexes highlights a significant disparity, with three males affected for every one female with the disease (1, 4). The prognosis of the disease is multifaceted and a function of environmental, genetic, epigenetic, and other variables (5–8). Accordingly, the study of tertiary prevention measures that diminish the risk of progression and prolong survival in this cancer population has become increasingly relevant in HNSCC.

Clinical and lifestyle factors influence the disease course and progression in HNSCC. Positive HPV serology, compared to negative HPV serology status, is associated with enhanced survival and a better response to treatment (3). There is mixed evidence of a deleterious effect of continued smoking and survival, although most studies ascribe a negative role of smoking, particularly in combination with continued drinking (9–12). Moreover, gender and race/ethnicity have been found to contribute to survival in HNSCC with significant disparities in certain race and gender combinations, particularly in non-white populations (13).

The relationship between dietary intake and primary prevention, or the mitigation of incident HNSCC, has been studied, albeit to a limited extent. Nonetheless, a consistent finding across studies has been the inverse relationship between fruit, vegetable, and antioxidant consumption and risk of HNSCC (14–17). Indeed, the World Cancer Research Fund/American Institute for Cancer Research (WCRF/AICR) Third Export Report from the Continuous Update Project (CUP) highlights limited although favorable evidence for a healthful diet, particularly non-starchy vegetables and coffee, in minimizing the risk of HNSCC (18). The literature concerning the role of dietary intake on pertinent outcomes following an HNSCC diagnosis is also sparse, although the limited evidence suggests that several dietary factors may influence prognosis in HNSCC. The University of Michigan Head and Neck Specialized Program of Research Excellence (UM-SPORE) is the only prospective survival cohort study of HNSCC that, to our knowledge, collected comprehensive and detailed dietary data on its participants at multiple points across the disease continuum. There have been several associations reported between modifiable lifestyle factors and cancer-related outcomes in patients from this study (19–23). Particularly, associations between baseline dietary fruit or vegetable consumption and nutrients associated with their intake and risk of HNSCC or prognosis of HNSCC have demonstrated a consistent trend that highlights a beneficial role of these food groups (24, 25). Concerning the role of symptoms and treatment on diet quality, nutrition impact symptoms (NIS), secondary to the disease or chemoradiotherapy, may affect the survivors’ ability to maintain their normal dietary pattern or adopt more healthful patterns (26, 27).

Nevertheless, the field of nutritional epidemiology has steadily shifted toward analyses that emphasize dietary patterns rather than single nutrients or food groups (28, 29). A recent analysis of these cohort data found that *a posteriori* dietary patterns measured at baseline, i.e., those extracted from data with multivariate statistical procedures, predicted survival in this cohort (23). The question of whether *a priori* diet quality indices, which measure adherence to a set of established dietary guidelines and are more generalizable to the study or general population, predict survival has never been broached in the context of the HNSCC population, although the relationship between *a priori* diet quality indices and the presence of NIS 1-year following diagnosis has previously been investigated in this cohort (30). Furthermore, the dynamic nature of dietary intake is rarely a focus of these analyses, and dietary variables are implemented into analytical models as static or baseline values only. Thus, the purpose of this analysis was to evaluate the performance of six *a priori*-defined diet quality indices, thrice measured annually from the start of the patient’s treatment protocol, by ascertaining differences related to survival for those diagnosed with malignant HNSCC.

## Materials and Methods

### Study Population

The UM-SPORE is a longitudinal cohort study of newly diagnosed HNSCC patients from the University of Michigan Hospital system. Recruitment took place between November 2008 through October 2014. The exclusionary criteria included: (i) being less than 18 years of age, (ii) being pregnant, (iii) being a non-English speaker, (iv) having a previously diagnosed mental health disorder, (v) previous or current diagnosis of a malignant tumor in the non-upper aerodigestive tract, or (vi) having a previous diagnosis with another form of primary HNSCC within the last 5 years. Data collection began before patients underwent their first course of treatment for HNSCC. Baseline (pretreatment) measures included dietary data collected via the self-administered and semi-quantitative 2007 Harvard Food Frequency Questionnaire (FFQ). Behavioral, social, and other health-related characteristics were collected via survey at baseline. These surveys assessed lifestyle variables such as smoking and drinking habits, sleep quality and quantity, mental health, physical activity, among other factors. Study participants were followed longitudinally with annual medical chart reviews being held to ascertain any medical or treatment history changes.

Among the 550 consenting respondents with available baseline dietary data, participants with tumors in anatomical sites other than the oral cavity, tongue, oropharynx, larynx, and hypopharynx (*n* = 45), missing full pages in their baseline FFQ (*n* = 17), having greater than 70 blank responses among one of the three FFQs administered (*n* = 7), and missing baseline covariates used in the analysis (*n* = 7) were excluded. Lastly, those reporting > 5,000 kcal on any of the three administered FFQs were excluded assuming the implausibility of such extreme intake (31). The final analytic cohort comprised 468 participants with baseline records, 329 participants with records 1-year postdiagnosis, and 261 participants with records present 2-years-postdiagnosis. All study protocols complied with the Helsinki Declaration of 1975 and were approved by the University of Michigan Institutional Review Board (IRB approval number for obtaining and analyzing the data, is HUM00042189).

### Explanatory Variables

Diet quality index scores for each of the following six previously defined indices were computed: The Alternate Healthy Eating Index (HEI)-2010 (AHEI-2010), the Alternate Mediterranean Diet Index (aMED), the Dietary Approaches to Stop Hypertension (DASH), and three low-carbohydrate diet composite scores: An overall low-carbohydrate index, an animal-based iteration, and a plant-based iteration. The operationalization of these indices will be described succinctly. All scores were assimilated using dietary intake data amassed *via* the 2007 Harvard Food Frequency Questionnaire (32).

The AHEI-2010 was proposed as an alternative to the HEI, a diet quality index that was developed around the Dietary Guidelines for Americans. The operational definition implemented in this analysis was that proposed by Chiuve et al. and relies on thresholds of absolute values of intake rather than population-based (i.e., quantile-based) cut-offs as the other indices in this analysis implement (33). Like the aMED index, AHEI-2010 includes an alcohol component. However, in contrast to the other indices, AHEI-2010 emphasizes dietary fat quality, rewarding polyunsaturated fat (PUFA) intake, and castigating trans-fat intake. The theoretical minimum and maximum values for this index are 0 and 110, respectively.

The aMED Index is based on the operational definition given by Fung et al. (34). The index is a proxy indicator of adherence to a Mediterranean-style diet that is conceptualized around nine food intake components: vegetables, legumes, fruits, nuts, cereals, red or processed meats, fish, alcohol, and the ratio of monounsaturated: saturated (MUFA: SFA) fat intake. Briefly, the scores were calculated by giving participants ranking higher than the median intake for a given “good” food component a score of “1.” The opposite scoring scheme was implemented for “bad” food components (i.e., lower intakes were rewarded). Alcohol intakes between 5 and 15 g/day were designated a score of “1” for the alcohol component. The final calculated scores ranged from 0 to 9.

The DASH Index is based on the criteria proposed by Fung et al. (35). The score ranges from 0 to 40 and is based on quintile rankings across eight food categories: Fruits, vegetables, nuts/legumes, low-fat dairy products, whole grains, sodium, red and processed meats, and sugar-sweetened beverages. The DASH protocol emphasizes fruits, vegetables, whole grains, low-fat dairy, nuts, and legumes and discourages high intakes of red meat, sugar-sweetened beverages, and other processed products (36).

Lastly, a series of low-carbohydrate indices, developed by Halton et al. were included in the analysis (37). Several lines of evidence, originating primarily from preclinical data, have argued a beneficial role for low-carbohydrate diets in the context of cancer prognosis (38, 39). The elevated interest in low-carbohydrate and very low-carbohydrate diets stems from promising evidence showing mitigation of tumor growth in mice, other animal models, as well as an analysis of this cohort that found an association between higher carbohydrate consumption and worse survival (20, 40). The operational definition of this index was based on rankings (deciles) for the percentage of calories from each of the fats, protein, and carbohydrates. The index rewarded higher intakes of fat and protein and lower intakes of carbohydrates with a theoretical minimum score of 0 and a maximum of 30. Animal-based and plant-based iterations of this index were also operationalized by using the percentage of calories from fat and percentage of calories from protein from animal or vegetable sources, respectively, in the place of total fat and total protein. We generated alluvial plots to visualize changes in a subject’s quintile ranking across time for all diet quality indices evaluated. The exact algorithms for computing these are included as figures in Supplementary Material.

### Modeling Approach

The associations between each diet quality index and the risk of mortality were assessed with three different survival models. This approach was adopted from previous analyses involving marginal structural models. It involved fitting a marginal structural Cox Proportional Hazards model, an unweighted Cox proportional hazards model with time-updated indicators of the diet indices, and a conventional time-independent Cox proportional hazards model with only baseline dietary intake values (41–44). Time-dependent, or time-varying, confounding arises when time-varying or baseline covariates confound the relationship between other time-varying covariates of interest and an outcome, leading to biased estimates of model parameters (45). Several modeling approaches exist for dealing with time-dependent confounding, and we used marginal structural models applied to time-varying Cox models, described extensively in the epidemiology and biostatistics literature, to probe our research questions (41, 42, 45, 46). The comparison of estimates from a weighted Cox proportional hazards model (i.e., the marginal structural model) to an unweighted model allows for evaluating the presence of time-dependent confounding, whereas the comparison of the marginal structural model to a standard Cox proportional hazards model allows for evaluation of whether misclassification bias, for instance, was an issue for a standard model considering only a static measure of dietary intake. In total, data from three follow-up visits were used for the analyses involving a time-updated measure of dietary intake: Baseline (pretreatment), 1-year postdiagnosis, and 2-years postdiagnosis.

Marginal structural models (MSMs) employ inverse probability weighting for treatment (IPTW) and censor status (IPCW). The former involves computing weights by taking the inverse of the probability that a subject receives their observed treatment, in this case, their quantile of intake for a diet index, conditional on their covariate values and previous treatment history. In contrast, the latter involves the inverse of the probability a subject is censored or lost to follow-up at the end of a given administrative time interval given their covariate history. These weights were calculated using the methodology described by Robins and Hernan (41, 42). The computation of the stabilized IPTW is represented in Equation *i*. In this notation, we use *i* to denote a subject, *k* to denote a realized measurement instance with a value ≤ *t*, which denotes the study visit for which the weight is being calculated. Moreover, lowercase symbols indicate realized values, whereas uppercase symbols are random variables. Thus, the computation proceeds in the following manner:


(i) $$s\,w_{i\,t}^{I\,P\,T\,W}=\prod_{k=1}^{t}\frac{P\left(A_{i\,k}=a_{i\,k}|{\bar{A}}_{i\,\left(k-1\right)}={\bar{a}}_{i\,\left(k-1\right)},V=v_{i}\right)}{P\left(A_{i\,k}=a_{i\,k}|{\bar{A}}_{i\,\left(k-1\right)}={\bar{a}}_{i\,\left(k-1\right)},V=v_{i},{\bar{L}}_{i\,k}={\bar{l}}_{i}\right)}$$

where, *A*_*ik*_ = *a*_*ik*_ denotes the observed treatment history of the *i*th subject at time *k*, which represents one of the five quintiles of the dietary exposure, ${\bar{A}}_{i\,\left(k-1\right)}={\bar{a}}_{i\,\left(k-1\right)}$ represents the observed treatment history (which is a vector of values constructed as a lagged variable representing the value for the last person-year), *V = v_i_* denotes the vector of observed values for baseline covariates, and ${\bar{L}}_{i\,k}={\bar{l}}_{i}$ is the vector of observed values for additional time-varying covariates. The stabilized IPCW are similarly computed by the following:


(ii) $$s\,w_{i\,t}^{I\,P\,C\,W}=\prod_{k=1}^{t}\frac{\begin{array}{c} P(C_{i\,k}=0|{\bar{C}}_{i\,\left(k-1\right)}={\bar{c}}_{i\,\left(k-1\right)},\\ {\bar{A}}_{i\,\left(k-1\right)}={\bar{a}}_{i\,\left(k-1\right)},V=v_{i}) \end{array}}{\begin{array}{c} P(C_{i\,k}=0|{\bar{C}}_{i\,\left(k-1\right)}={\bar{c}}_{i\,\left(k-1\right)},{\bar{A}}_{i\,\left(k-1\right)}={\bar{a}}_{i\,\left(k-1\right)},\\ V=v_{i},{\bar{L}}_{i\,\left(k-1\right)}={\bar{l}}_{i\,\left(k-1\right)}) \end{array}}$$

where, in the numerator, we obtain the probability of remaining uncensored for each person-year, *k*, conditioned on the prior observed censoring history (${\bar{C}}_{i\,\left(k-1\right)}={\bar{c}}_{i\,\left(k-1\right)}$), which was formulated as a lagged variable, the observed value of the treatment (${\bar{A}}_{i\,\left(k-1\right)}={\bar{a}}_{i\,\left(k-1\right)}$), and the baseline covariates (*V = v_i_*). For the denominator, values of the additional time-varying covariates at the preceding visit are included. The final and stabilized weights are computed by taking the product in Equation *iii*.


(iii) $$s\,w_{i\,t}=s\,w_{i\,t}^{I\,P\,T\,W}*s\,w_{i\,t}^{I\,P\,C\,W}$$

Thus, the denominator of the product of $s\,w_{i\,t}^{I\,P\,T\,W}$ and $s\,w_{i\,t}^{I\,P\,C\,W}$ represents the conditional probability that a subject receives their treatment history and censoring history up to time *t* (42).

### Model Specification

Weight models were specified with the incorporation of restricted cubic splines, using three knots, for each of the time-varying confounders (i.e., for time-updated BMI and calories) as previously suggested for achieving the correct model specification (47). Weight models without splines were first evaluated for their behavior (i.e., means, median, and range) before the addition of splines, to which they were subsequently compared and found to be better-behaved. Moreover, truncated weights mitigate the influence of observations harboring extreme weights, yielding more precise standard error estimates with tighter confidence intervals, though progressive truncation may result in biased model estimates (47). Therefore, a decision was made to truncate weights at the 98th percentile per previous recommendations (47). Subjects with weights greater than or equal to the 98th percentile for the stabilized weights were assigned that value. The marginal structural Cox model was 408 specified in the *svycoxph* function (from the *survey* package) as:


(iv) $$\lambda_{i}\left[t|{\bar{A}}_{i\,t},V_{i}\right]=\lambda_{0}\left(t\right)\text{exp}\left(\beta_{1}A_{i}\left(t\right)+\beta_{2}V_{i}+\beta_{3}A_{i}\left(t-1\right)\right)$$

where, the hazard of the *i^th^* subject at time *t* is conditioned on exposure history at time *t*, the vector of baseline covariates, *V_i_*, and the past exposure history, *A*_*i*_(*t*−1), of the *i^th^* subject (48–50). β_1_ is the causal log hazard rate for the time-varying exposure of interest (note that there were four coefficients as this variable was dummy coded in the analysis using an ordinal five level factor for each dietary index) (41, 51). The weighted and unweighted survival models included the time-updated dietary exposure indicator (in quintiles), the diet index score measured at the previous encounter, and the baseline covariates: age, sex, HPV status, tumor site, cancer stage, treatment modality, Adult Co-Morbidity Evaluation (ACE-27), highest education level attained, smoking status, baseline body mass index (BMI), and baseline caloric intake. A test for linear trend across index quintiles was modeled similarly. A time-updated trend variable was generated for each participant in each of the *t* timepoints. Participants were assigned the median value of their respective quintile of intake for each of the diet quality indices. This variable was subsequently modeled as a continuous and time-updated covariate. We also assessed linear fit with the dietary exposures modeled continuously and scaled by their respective standard deviations using linear and quadratic terms. Lastly, we modeled each continuous diet index score with restricted cubic splines that used five knots. Standard Cox proportional hazards models using only baseline values of the diet indices were fit and adjusted only for baseline covariates.

Effect modification was evaluated across *a priori*-selected baseline characteristics: cancer stage, HPV status, and tumor site by including interaction terms in the MSMs with the scaled continuous index variables noted above and using the likelihood ratio test (42, 52, 53).

### Covariates

The covariates in both weight and analytical models included age (continuous), sex (categorized dichotomously), smoking status (never, former, and current), highest education level attained (less than or equal to high school or greater than high school), HPV status (positive, negative, and equivocal/unknown), tumor stage (categorized as 0–II or III–IV), ACE-27 (categorized as none, mild, moderate, and severe), treatment modality (surgery alone, surgery and adjuvant radiation or chemoradiation, radiation alone, chemoradiation alone, chemotherapy alone, and palliative or unknown treatment), and tumor site (larynx/hypopharynx, oral cavity, or oropharynx). Additionally, BMI and caloric intake were included as continuous time-varying covariates. To mitigate collinearity, no analyses adjusted for drinking status given a significant correlation with smoking status. Likewise, the AHEI-2010 and aMED analyses contain an alcohol intake component in their calculation, which would lead to collinearity.

### Outcomes: All-Cause Mortality and Cancer-Specific Mortality

The events of analytic interest, deaths from all causes, and cancer-specific mortality were documented using either the Social Security Death Index or the LexisNexis, updates to medical and survey data at each of the follow-up time-points, and through notification from family, other physicians, or medical record reviews. The survival time was initiated with a start date corresponding to the date of diagnosis, and an administrative censoring date of 3 years following the initial follow-up study visit was used. We analyzed only the initial 3 years of observation, given that the participants only completed the set of three FFQs during this window, and we did not want to extrapolate the dietary intake data into the ensuing years. Individuals with missing dates for any of the three visits included in the analysis but who returned for a subsequent visit were assigned the date corresponding to the median survival time added or subtracted to a documented date in order to generate the length of the missing period. Participants were censored if they experienced loss to follow-up at any point in time throughout the length of the study period if they had reported an invalid social security number that would preclude study investigators from formally adjudicating death should it have occurred, or if they failed to experience the event of interest during the allotted study timeframe.

### Missing Data, Imputation, and Sensitivity Analyses

The longitudinal nature of these data led to missing values along the follow-up trajectory of select subjects. We dealt with missing follow-up visits in the following manner: participants who did not participate in either of the two follow-up FFQ measurements but who were otherwise tracked longitudinally were treated as lost to follow-up. That is, they were censored at the end of the first interval period for which they had pretreatment FFQ data available and did not have any subsequent visits imputed (*n* = 78). Individuals with complete FFQ data for at least two visits but who missed only one of those three visits FFQs had their trajectory mean, using their two available measurements, imputed for the diet index scores and caloric intake (*n* = 45) (54, 55). Thus, the imputation procedure imputed values for 45 (4.4%) records out of a total of 1,018 records used in the analysis. In addition, there were 12 subjects with missing follow-up BMI measures from the third visit. All 12 of these subjects were also those with missing dietary data imputed. In order to assess any bias introduced by the imputation process, we censored all individuals with imputed records after the first follow-up study visit and re-ran our analysis to assess any changes in parameter estimates. All the code to reproduce this analysis may be found at: https://github.com/cmainov/ap_Indices_Surv_HNSCC.

## Results

### Descriptive Analysis

The study culminated in the analysis of 1,018 person-years with 93 deaths from all causes and 74 cancer-related deaths. The epidemiologic characteristics of the study sample are detailed in Table 1. At study entry, the majority of participants reported identifying as males (75.2%), current or former smokers (71.6%), and as non-Hispanic white (95.1%). There were appreciable differences in the baseline study characteristics among high and low scorers for the AHEI-2010, aMED, and DASH indices. Notably, there was a more significant proportion of females in the highest compared to the lowest quantiles of those indices. The attained education status was greater among those with higher scores in the AHEI-2010, aMED, and DASH indices than those with lower scores. For the low-carbohydrate indices, the proportion of college-educated subjects in those with higher scores was commensurate to those with lower scores. However, the plant-based low-carbohydrate index showed a larger discrepancy compared to the other two variants of this index, with a greater proportion of subjects attaining a higher education status. High scorers in the AHEI-2010, aMED, DASH indices, and the plant-based low-carbohydrate index exhibited lower proportions of current smokers and higher proportions of former smokers than low scorers. There were no apparent differences in these trends across high and low scorers of the low-carbohydrate index, and there was a greater relative proportion of current smokers and a lower proportion of former smokers in the high scoring group of the animal-based low-carbohydrate index compared to the low-scoring group. The mean BMI was commensurate in higher and lower scorers across all diet indices.

**TABLE 1:** Demographic, clinical, and behavioral baseline characteristics of the study participants in the overall study sample (*n* = 468) and across high and low scores (split at the median) of the diet quality indices.

Characteristic		AHEI-2010		aMED		DASH		Low-carbohydrate (LC)		Animal-based LC		Plant-based low-LC	
							
	Survivors # (%)	M1 (*n* = 234)	M2 (*n* = 234)	M1 (*n* = 199)	M2 (*n* = 269)	M1 (*n* = 217)	M2 (*n* = 251)	M1 (*n* = 229)	M2 (*n* = 239)	M1 (*n* = 216)	M2 (*n* = 252)	M1 (*n* = 205)	M2 (*n* = 263)
**Age (y)**													
Mean (SD)	61.1 (11.2)	60 (11.6)	62.3 (10.7)	59.9 (11.3)	62.1 (11.0)	59.2 (10.9)	62.8 (11.1)	61.1 (11.6)	61.2 (10.8)	61.5 (11.8)	60.8 (10.7)	59.9 (11.3)	62.1 (11.0)
Min/max	25/95	25/95	30/92	25/87	30/95	25/93	30/95	25/95	29/93	25/95	29/93	25/90	30/95
**Sex**													
Male	352 (75.2)	188 (80.3)	164 (70.1)	155 (77.9)	197 (73.2)	177 (81.6)	175 (69.7)	171 (74.7)	181 (75.7)	161 (74.5)	191 (75.8)	153 (74.6)	199 (75.7)
Female	116 (24.8)	46 (19.7)	70 (29.9)	44 (22.1)	72 (26.8)	40 (18.4)	76 (30.3)	58 (25.3)	58 (24.3)	55 (25.5)	61 (24.2)	52 (25.4)	64 (24.3)
**Education**													
High school or less	160 (34.2)	100 (42.7)	60 (25.6)	87 (43.7)	73 (27.1)	95 (43.8)	65 (25.9)	81 (35.4)	79 (33.1)	76 (35.2)	84 (33.3)	79 (38.5)	81 (30.8)
Some college or more	308 (65.8)	134 (57.3)	174 (74.4)	112 (56.3)	196 (72.9)	122 (56.2)	186 (74.1)	148 (64.6)	160 (66.9)	140 (64.8)	168 (66.7)	126 (61.5)	182 (69.2)
**Race**													
Non-Hispanic white	445 (95.1)	221 (94.4)	224 (95.7)	186 (93.5)	259 (96.3)	203 (93.5)	242 (96.4)	218 (95.2)	227 (95.0)	207 (95.8)	238 (94.4)	196 (95.6)	249 (94.7)
Other	23 (4.9)	13 (5.6)	10 (4.3)	13 (6.5)	10 (3.7)	14 (6.5)	9 (3.6)	11 (4.8)	12 (5.0)	9 (4.2)	14 (5.6)	9 (4.4)	14 (5.3)
**BMI**													
(kg/m^2^) mean (SD)	27.7 (5.8)	27.6 (6.4)	27.7 (5.2)	27.7 (6.0)	27.6 (5.7)	27.4 (6.3)	27.9 (5.4)	27.1 (5.7)	28.2 (5.9)	27.4 (5.9)	27.9 (5.8)	27.7 (5.8)	27.6 (5.9)
**Site**													
Larynx/Hypopharynx	106 (22.6)	61 (26.1)	45 (19.2)	56 (28.1)	50 (18.6)	60 (27.6)	46 (18.3)	47 (20.5)	59 (24.7)	46 (21.3)	60 (23.8)	45 (22.0)	61 (23.2)
Oral cavity	170 (36.3)	87 (37.2)	83 (35.5)	73 (36.7)	97 (36.1)	77 (35.5)	93 (37.1)	87 (38.0)	83 (34.7)	77 (35.6)	93 (36.9)	85 (41.5)	85 (32.3)
Oropharynx	192 (41.0)	86 (36.8)	106 (45.3)	70 (35.2)	122 (45.4)	80 (36.9)	112 (44.6)	95 (41.5)	97 (40.6)	93 (43.1)	99 (39.3)	75 (36.6)	117 (44.5)
**Stage**													
0, I, II	144 (30.8)	64 (27.4)	80 (34.2)	60 (30.2)	84 (31.2)	59 (27.2)	85 (33.9)	75 (32.8)	69 (28.9)	70 (32.4)	74 (29.4)	68 (33.2)	76 (28.9)
III, IV	324 (69.2)	170 (72.6)	154 (65.8)	139 (69.8)	185 (68.8)	158 (72.8)	166 (66.1)	154 (67.2)	170 (71.1)	146 (67.6)	178 (70.6)	137 (66.8)	187 (71.1)
**HPV Status**													
HPV-negative	153 (32.7)	81 (34.6)	72 (30.8)	75 (37.7)	78 (29.0)	78 (35.9)	75 (29.9)	73 (31.9)	80 (33.5)	68 (31.5)	85 (33.7)	69 (33.7)	84 (31.9)
HPV-positive	77 (16.5)	36 (15.4)	41 (17.5)	30 (15.1)	47 (17.5)	30 (13.8)	47 (18.7)	40 (17.5)	37 (15.5)	42 (19.4)	35 (13.9)	33 (16.1)	44 (16.7)
Unknown/Equivocal	238 (50.9)	117 (50.0)	121 (51.7)	94 (47.2)	144 (53.5)	109 (50.2)	129 (51.4)	116 (50.7)	122 (51.0)	106 (49.1)	132 (52.4)	103 (50.2)	135 (51.3)
**ACE-27**													
None	124 (26.5)	62 (26.5)	62 (26.5)	48 (24.1)	76 (28.3)	55 (25.3)	69 (27.5)	59 (25.8)	65 (27.2)	58 (26.9)	66 (26.2)	50 (24.4)	74 (28.1)
Mild	227 (48.5)	102 (43.6)	125 (53.4)	88 (44.2)	139 (51.7)	100 (46.1)	127 (50.6)	109 (47.6)	118 (49.4)	98 (45.4)	129 (51.2)	101 (49.3)	126 (47.9)
Moderate	84 (17.9)	49 (20.9)	35 (15.0)	46 (23.1)	38 (14.1)	43 (19.8)	41 (16.3)	46 (20.1)	38 (15.9)	44 (20.4)	40 (15.9)	43 (21.0)	41 (15.6)
Severe	33 (7.1)	21 (9.0)	12 (5.1)	17 (8.5)	16 (5.9)	19 (8.8)	14 (5.6)	15 (6.6)	18 (7.5)	16 (7.4)	17 (6.7)	11 (5.4)	22 (8.4)
**Smoking status**													
Never	133 (28.4)	57 (24.4)	76 (32.5)	48 (24.1)	85 (31.6)	49 (22.6)	84 (33.5)	59 (25.8)	74 (31.0)	62 (28.7)	71 (28.2)	53 (25.9)	80 (30.4)
Current	170 (36.3)	104 (44.4)	66 (28.2)	89 (44.7)	81 (30.1)	95 (43.8)	75 (29.9)	84 (36.7)	86 (36.0)	72 (33.3)	98 (38.9)	82 (40.0)	88 (33.5)
Former	165 (35.3)	73 (31.2)	92 (39.3)	62 (31.2)	103 (38.3)	73 (33.6)	92 (36.7)	86 (37.6)	79 (33.1)	82 (38.0)	83 (32.9)	70 (34.1)	95 (36.1)

*Percentages may not add to 100% given rounding.*

*M1 denotes the lower half of the data (i.e., below the respective median index score).*

*M2 denotes the upper half of the data (i.e., above the respective median index score).*

**FIGURE 1:**
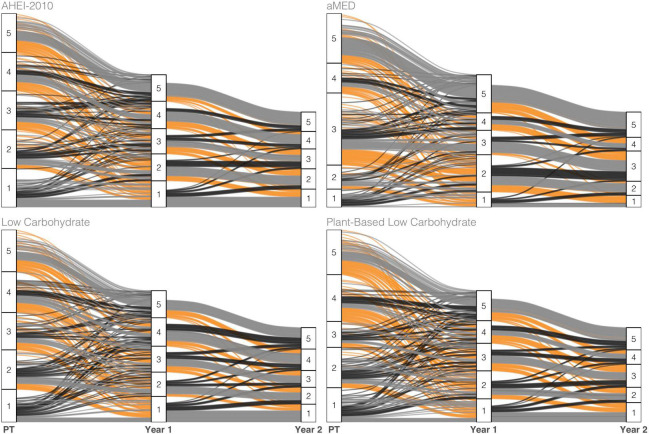
Alluvial plot tracking subject-specific diet index scores of select indices across the three measurement points—pretreatment (PT), 1 year, and 2 years post-diagnosis—of observation. The color scheme maps to three classes of change observed across adjacent years: Increase (black), no change (gray), or decrease (orange) in ranking.

Changes in diet index quintile over follow-up time are visualized with alluvial plots of select indices in Figure 1. Overall, the transiency in quintile ranking was greater in the lower quintiles (Q1-Q4) of the diet indices, while greater proportions of Q5 retained this ranking across time. The low-carbohydrate indices observed greater upward vertical mobility across time for subjects in Q1 at pretreatment than other indices. Also, changes did not appear to change substantially from the year-one to year-two visits in all indices, thus suggesting greater stability during this time window.

### Stabilized Weights and MSMs

**FIGURE 2:**
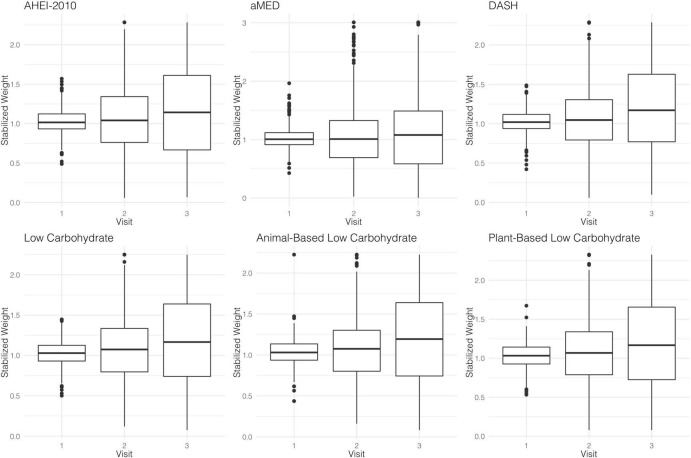
Distributions of diet quality index-specific weights at each study visit.

**FIGURE 3:**
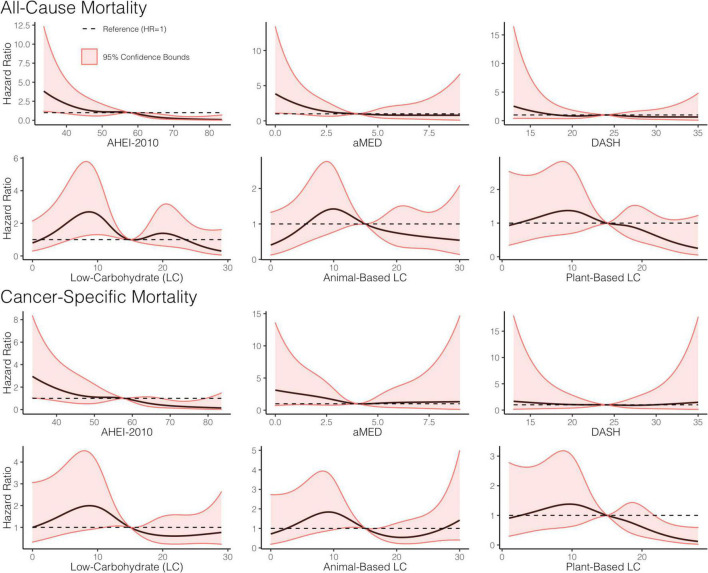
Dose-response relationship between adherence to *a priori* diet quality indices, all-cause, and cancer-specific mortality. Restricted cubic splines, with five knots, were used to construct smooth curves from models that evaluated the indices as continuous variables and were weighted using the weights from the marginal structural models. Hazard ratios (HR) were computed with the referent set as the hazard for the median index score. A dashed line showing HR = 1 is included for reference. All models adjusted for baseline age, sex, tumor site, stage, human papillomavirus (HPV) status, treatment modality, ACE-27, the highest level of education attained, index score measured at the previous encounter, smoking status, caloric intake, and body mass index (BMI).

The parameter estimates for distributions of stabilized weights computed across diet quality indices and study visits are documented in Table 2 and Figure 2. Overall, the weights appeared well-behaved, with all weight means approximately converging on a value of one and presenting with narrow ranges, both of which provide corroborating evidence of correct model specification (47, 56). Weighted and adjusted results from MSM’s for each of the dietary indices examined were mixed (Table 3). The highest consumption along AHEI-2010 throughout the 3 years of observation was significantly associated with a 93% decrease in the risk of all-cause mortality compared to the lowest quintile of adherence. A significant linear trend was also observed across the quintiles of this pattern (*p*_trend_ = 0.04), and the significant linear association further corroborated this observation observed when this index was modeled continuously. Each standard deviation increase in adherence to the AHEI-2010 was significantly associated with a 60% reduction in the risk of all-cause mortality, and there was no evidence of a non-linear relationship (*p* = 0.43) (Table 3 and Figure 3).

**TABLE 2:** Descriptive statistics for stabilized weights computed using weight models.

Index	Visit	Min	Max	Mean	Median
AHEI-2010	1	0.49	1.57	1.02	1.02
AHEI-2010	2	0.06	2.28	1.08	1.04
AHEI-2010	3	0.07	2.28	1.17	1.14
aMED	1	0.43	1.96	1.03	1.01
aMED	2	0.03	3.01	1.09	1.01
aMED	3	0	3.01	1.18	1.08
DASH	1	0.42	1.49	1.02	1.02
DASH	2	0.06	2.29	1.07	1.05
DASH	3	0.10	2.29	1.21	1.17
Low carbohydrate	1	0.50	1.45	1.03	1.03
Low carbohydrate	2	0.12	2.25	1.09	1.07
Low carbohydrate	3	0.07	2.25	1.21	1.17
Animal-based low carbohydrate	1	0.44	2.22	1.03	1.03
Animal-based low carbohydrate	2	0.16	2.22	1.08	1.07
Animal-based low carbohydrate	3	0.08	2.22	1.21	1.19
Plant-based low carbohydrate	1	0.53	1.67	1.03	1.03
Plant-based low carbohydrate	2	0.08	2.33	1.08	1.07
Plant-based low carbohydrate	3	0.08	2.33	1.22	1.17

**TABLE 3:** Hazard ratios^†^ (HRs) and 95% CIs for all-cause mortality from both marginal structural models and unweighted models across each dietary index.

Diet quality index	Model^†^	Q1	Q2	Q3	Q4	Q5	*p*_trend_	*p*_*Q5*–*Q1*_	HR^a^_continuous_	*p^b^_quadratic_*
AHEI-2010	MSM	1.00	0.38 (0.13–1.12)	0.41 (0.10–1.76)	0.37 (0.06–2.18)	0.07 (0.01–0.43)**	0.04*	<0.01**	0.40 (0.23–0.70)**	0.43
	Unweighted	1.00	0.33 (0.13–0.79)*	0.35 (0.13–0.94)*	0.29 (0.08–1.08)	0.09 (0.02–0.38)**	0.02*	<0.01**	0.46 (0.29–0.74)**	0.39
aMED	MSM	1.00	0.34 (0.10–1.17)	0.44 (0.10–1.95)	0.28 (0.04–2.00)	0.52 (0.05–5.06)	0.77	0.58	0.78 (0.30–2.06)	0.02*
	Unweighted	1.00	0.57 (0.20–1.66)	0.59 (0.18–1.96)	0.37 (0.10–1.36)	0.41 (0.09–1.85)	0.73	0.24	0.68 (0.37–1.25)	0.07
DASH	MSM	1.00	0.61 (0.21–1.80)	1.15 (0.33–4.03)	0.88 (0.12–6.24)	0.73 (0.06–8.81)	0.77	0.81	0.69 (0.27–1.75)	0.31
	Unweighted	1.00	0.97 (0.33–2.83)	1.49 (0.47–4.73)	1.12 (0.24–5.19)	1.05 (0.14–7.67)	0.74	0.96	0.77 (0.36–1.61)	0.28
LC^c^	MSM	1.00	0.57 (0.28–1.18)	0.26 (0.11–0.61)**	0.57 (0.24–1.35)	0.41 (0.11–1.55)	0.03*	0.19	0.85 (0.62–1.17)	0.04*
	Unweighted	1.00	0.59 (0.29–1.20)	0.31 (0.14–0.65)**	0.48 (0.21–1.07)	0.32 (0.10–1.04)	< 0.01**	0.06	0.81 (0.60–1.09)	0.03*
Animal-based LC	MSM	1.00	1.15 (0.44–3.03)	1.44 (0.50–4.15)	0.61 (0.21–1.77)	1.23 (0.36–4.19)	0.24	0.75	1.00 (0.70–1.42)	0.05
	Unweighted	1.00	1.08 (0.45–2.56)	1.18 (0.42–3.31)	0.52 (0.17–1.58)	0.86 (0.27–2.77)	0.06	0.81	0.90 (0.63–1.28)	0.04*
Plant-based LC	MSM	1.00	0.34 (0.12–0.94)*	0.37 (0.13–1.08)	0.37 (0.13–1.10)	0.29 (0.07–1.14)	0.45	0.08	0.71 (0.50–1.01)	0.05
	Unweighted	1.00	0.61 (0.20–1.91)	0.56 (0.20–1.53)	0.60 (0.22–1.61)	0.48 (0.16–1.45)	0.55	0.19	0.81 (0.60–1.08)	0.04*

*There were 1,018 person-years of contributions used in this analysis and 93 documented deaths.*

**p < 0.01.

*p < 0.05.

*^†^All models adjusted for baseline age, sex, tumor site, stage, HPV status, treatment modality, ACE-27, highest level of education attained, index score measured at the previous encounter, smoking status, caloric intake, and BMI.*

*^a^Hazard ratio (HR) corresponding to a standard deviation increase in the diet index score.*

*^b^Wald test p-value for a quadratic polynomial term.*

*^c^Low carbohydrate (LC).*

**FIGURE 4:**
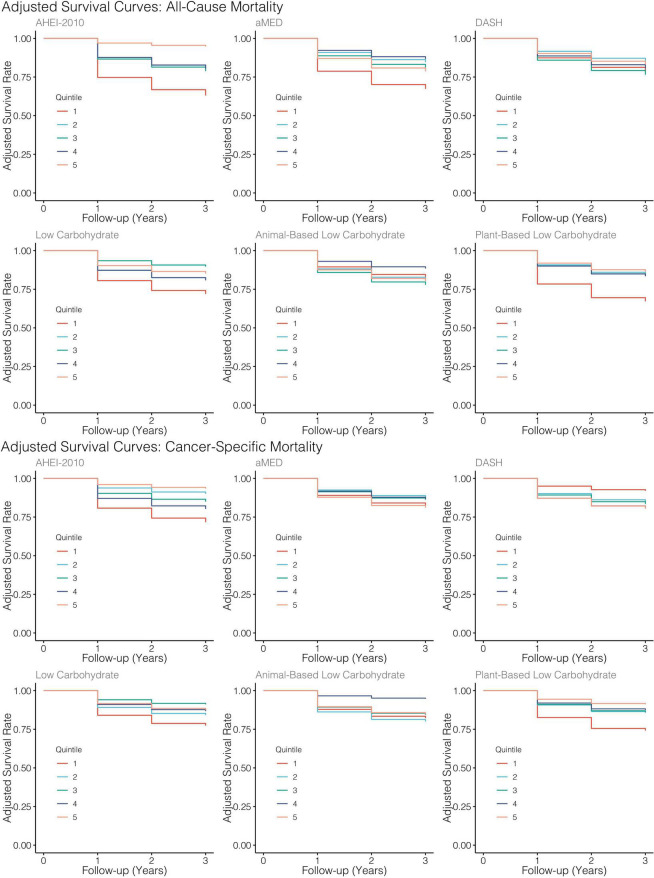
Adjusted survival curves generated from MSMs for each diet quality index. All models adjusted for baseline age, sex, tumor site, stage, HPV status, treatment modality, ACE-27, the highest level of education attained, index score measured at the previous encounter, smoking status, caloric intake, and BMI.

Similarly, higher scores along the low-carbohydrate and plant-based low-carbohydrate indices were associated with a significant 59 and 71%, respectively, decreased risk of all-cause mortality though these parameter estimates for the dummy-coded fifth quintile of adherence failed to meet the threshold for statistical significance. There was a significant downward trend observed across the quintiles of the low-carbohydrate index (*p* = 0.03) and, likewise, when modeled as continuous variables, each SD increase in the low-carbohydrate index and the plant-based low-carbohydrate index were associated with 15 and 29% reductions in risk of all-cause mortality, respectively, though these estimates were also both non-significant at the α = 0.05 level. Upon further investigation, it appeared that there was evidence of a parabolic relationship in all iterations of the low-carbohydrate indices although the restricted cubic splines analysis suggested significant non-linearity (Table 3 and Figure 3). A significant inverse association further supported this finding observed in the third quintile, relative to the first quintile, of the low-carbohydrate index. Notably, when compared to the other low-carbohydrate indices, the animal-based low-carbohydrate index was not associated with the risk of all-cause mortality, and parameter estimates across all models fitted with this index were close to the null value of one and non-significant. Overall, no significant associations were found for the aMED and DASH diet quality indices. The hazard ratio estimates for the highest quintiles of intake were generally suggestive of inverse relationships, though these estimates were unstable with wide confidence intervals. Nevertheless, it was apparent that a quadratic polynomial fit may be appropriate for the aMED index. Upon evaluation of the restricted cubic spline analysis, it appeared that, overall, there was no apparent association between the aMED index and all-cause mortality (Figure 3). Adjusted survival curves were generated from the MSMs (Figure 4) and visually depicted the relationships ascertained above in models considering ordinal versions of the diet quality index scores. Again, we observed a clear separation between Q5 of the AHEI-2010 from all other quintiles of that index.

When considering cancer-specific mortality (Table 4), there was, again, an inverse association between the AHEI-2010 and the risk of cancer-specific mortality. The parameter estimates, in this regard, were similar to those from all-cause mortality, with Q5 bearing an 85% reduction in the risk of cancer-specific mortality compared to Q1, although non-significant, and there was also a significant linear trend observed across the quintiles of adherence (*p* = 0.04). An SD increase in adherence to this index was associated with a significant 48% reduction in the risk of cancer-specific mortality. The low-carbohydrate index was not significantly associated with cancer mortality, though the parameter estimates in most models suggested an inverse association. When evaluating the quintile-specific estimates and the fact that there was no significant evidence of a linear relationship or a quadratic one, it became apparent that the evidence was not particularly strong for this index. There was a non-significant inverse association between the plant-based low-carbohydrate index and cancer-specific mortality, with the highest quintile showing a 75% reduction in the risk of cancer-specific mortality compared to the lowest quintile. However, each standard deviation increase in this index score was significantly associated with a 36% reduction in the risk of cancer-specific mortality. Again, there was evidence that a non-linear transformation may best model this relationship and the restricted cubic splines analysis, again, supported these results (Figure 3). Notably, in comparing these cause-specific mortality curves to those from all-cause mortality (Figure 3), we found that the relationships remained strong for the AHEI index and became stronger for the plant-based low-carbohydrate index, and it was apparent that the other indices did not have pronounced associations. In general, for both outcomes of all-cause and cancer-specific mortalities, the estimates from MSMs were similar to those from unweighted models, although there were specific instances where weighting either mitigated the effect sizes or amplified them, albeit not by substantial quantities.

**TABLE 4:** HRs^†^ and 95% CIs for cancer-specific mortality from both marginal structural models and unweighted models across each dietary index.

Diet quality index	Model^†^	Q1	Q2	Q3	Q4	Q5	*p*_trend_	*p*_*Q5*–*Q1*_	HR^a^_continuous_	*p^b^_quadratic_*
AHEI-2010	MSM	1.00	0.25 (0.06–1.05)	0.42 (0.07–2.49)	0.60 (0.11–3.18)	0.15 (0.02–1.07)	0.04*	0.06	0.52 (0.31–0.88)*	0.52
	Unweighted	1.00	0.25 (0.08–0.80)*	0.29 (0.08–1.02)	0.51 (0.13–1.95)	0.13 (0.03–0.66)*	0.04*	0.01*	0.54 (0.35–0.84)**	0.51
aMED	MSM	1.00	0.63 (0.15–2.74)	0.75 (0.11–5.14)	0.71 (0.08–6.36)	1.14 (0.09–14.40)	0.35	0.92	1.10 (0.39–3.11)	0.03*
	Unweighted	1.00	1.02 (0.36–2.87)	0.83 (0.22–3.16)	0.58 (0.13–2.60)	0.57 (0.10–3.20)	0.74	0.52	0.75 (0.36–1.59)	0.08
DASH	MSM	1.00	2.25 (0.77–6.58)	2.52 (0.44–14.58)	3.16 (0.22–46.14)	3.18 (0.11–89.55)	0.96	0.50	1.10 (0.27–4.40)	0.22
	Unweighted	1.00	2.61 (0.88–7.75)	2.64 (0.58–11.98)	2.23 (0.29–17.03)	2.15 (0.13–34.47)	0.82	0.59	0.92 (0.35–2.45)	0.24
LC^c^	MSM	1.00	0.61 (0.26–1.42)	0.30 (0.10–0.84)*	0.48 (0.17–1.39)	0.44 (0.10–1.90)	0.06	0.27	0.76 (0.50–1.14)	0.78
	Unweighted	1.00	0.55 (0.22–1.34)	0.30 (0.12–0.75)*	0.46 (0.17–1.26)	0.37 (0.10–1.47)	0.05	0.16	0.74 (0.52–1.06)	0.46
Animal-based LC	MSM	1.00	1.17 (0.37–3.72)	0.85 (0.31–2.29)	0.22 (0.05–0.93)*	0.82 (0.21–3.23)	0.25	0.78	0.84 (0.51–1.39)	0.68
	Unweighted	1.00	1.15 (0.41–3.23)	0.66 (0.23–1.95)	0.21 (0.05–0.86)*	0.71 (0.19–2.61)	0.20	0.61	0.81 (0.51–1.29)	0.92
Plant-based LC	MSM	1.00	0.45 (0.14–1.53)	0.43 (0.12–1.57)	0.38 (0.10–1.44)	0.25 (0.04–1.55)	0.12	0.14	0.64 (0.43–0.96)*	0.02*
	Unweighted	1.00	0.57 (0.19–1.77)	0.60 (0.21–1.71)	0.54 (0.18–1.60)	0.40 (0.11–1.53)	0.18	0.18	0.73 (0.55–0.97)*	0.01*

*There were 1,018 person-years of contributions used in this analysis and 74 documented deaths.*

**p < 0.01.

*p < 0.05.

*^†^All models adjusted for baseline age, sex, tumor site, stage, HPV status, treatment modality, ACE-27, highest level of education attained, index score measured at the previous encounter, smoking status, caloric intake, and BMI.*

*^a^Hazard ratio (HR) corresponding to a standard deviation increase in the diet index score.*

*^b^Wald test p-value for a quadratic polynomial term.*

*^c^Low carbohydrate (LC).*

### Secondary Analyses

Using interaction terms to assess for effect modification, we found, generally, no significant evidence for effect modification by cancer stage, tumor site, or HPV status in both outcomes of all-cause and cancer-specific mortalities for most of the indices (Supplementary Table 3). However, we did observe a significant aMED by tumor site interaction suggesting effect modification where a significant inverse association was present for subjects with tumors in the oropharynx but not in other site classes (Supplementary Table 4). When we used time-invariant measures of diet quality index adherence in standard Cox proportional hazards models that considered only baseline for these exposures, we found no significant associations between any diet quality indices and all-cause mortality or cancer-specific mortality (Table 5). The hazard ratio estimates, generally, appeared to trend in a similar direction as those observed from the time-varying Cox MSMs, although no estimates met the threshold for statistical significance. Additionally, the strongest associations from all-cause mortality models belonged to the low-carbohydrate index and the AHEI-2010, albeit non-significant. In the models evaluating cancer mortality, the associations became notably stronger for the AHEI-2010 and plant-based low-carbohydrate index. Finally, we also conducted two sensitivity analyses to evaluate any bias introduced by the imputation process. The results of this analysis (Supplementary Tables 1, 2) demonstrated that the parameter estimates were negligibly changed and, in general, mirrored those from the primary analysis. Moreover, neither the estimates’ significance nor interpretation was appreciably altered when we censored records of those subjects with imputed values for follow-up diet and BMI measures.

**TABLE 5:** HRs^†^ and 95% CIs for all-cause and cancer-specific mortality from traditional Cox Proportional Hazards Models (Cox PH) examining baseline index values only and employing administrative right-censoring at 3 years after diagnosis.

Diet quality index	Q1	Q2	Q3	Q4	Q5	*p*_trend_	*p*_*q5*–*q1*_
**All-cause mortality**							
AHEI-2010	1.00	1.16 (0.58–2.30)	1.20 (0.60–2.42)	0.76 (0.37–1.60)	0.72 (0.33–1.57)	0.24	0.41
aMED	1.00	0.88 (0.35–2.16)	0.59 (0.26–1.32)	0.88 (0.35–2.20)	0.74 (0.31–1.79)	0.99	0.51
DASH	1.00	0.78 (0.40–1.53)	0.92 (0.43–2.00)	0.65 (0.31–1.35)	0.79 (0.37–1.68)	0.44	0.53
Low carbohydrate	1.00	1.27 (0.64–2.53)	1.38 (0.68–2.78)	0.81 (0.36–1.79)	0.68 (0.32–1.46)	0.11	0.33
Animal-based low carbohydrate	1.00	1.11 (0.55–2.22)	1.31 (0.67–2.53)	1.01 (0.50–2.03)	0.55 (0.26–1.17)	0.10	0.12
Plant-based low carbohydrate	1.00	1.41 (0.69–2.90)	1.45 (0.66–3.17)	1.93 (0.97–3.84)	0.83 (0.38–1.78)	0.76	0.62
**Cancer-specific mortality**							
AHEI-2010	1.00	0.86 (0.41–1.80)	1.12 (0.53–2.36)	0.55 (0.24–1.26)	0.55 (0.23–1.31)	0.09	0.18
aMED	1.00	1.01 (0.39–2.66)	0.65 (0.27–1.58)	0.84 (0.29–2.40)	0.98 (0.37–2.57)	0.68	0.96
DASH	1.00	0.89 (0.44–1.82)	1.04 (0.45–2.38)	0.46 (0.20–1.08)	0.74 (0.32–1.70)	0.19	0.48
Low carbohydrate	1.00	1.82 (0.83–3.96)	1.65 (0.72–3.75)	0.65 (0.25–1.69)	0.95 (0.41–2.19)	0.20	0.91
Animal-based low carbohydrate	1.00	1.02 (0.47–2.21)	1.17 (0.56–2.44)	0.83 (0.37–1.85)	0.71 (0.33–1.53)	0.29	0.39
Plant-based low carbohydrate	1.00	1.16 (0.54–2.48)	1.84 (0.83–4.08)	1.52 (0.72–3.21)	0.59 (0.25–1.40)	0.34	0.23

**p < 0.01.

*p < 0.05.

*^†^All models adjusted for baseline age, sex, tumor site, stage, HPV status, treatment modality, ACE-27, highest level of education attained, smoking status, caloric intake, and BMI.*

## Discussion

Using time-updated, marginal structural models, we found that higher consumption along the AHEI-2010, two low-carbohydrate indices, and the aMED index throughout the first 3 years of observation was inversely associated with risks of all-cause mortality and cancer-specific mortality in a prospective survival cohort study of patients with HNSCC. There was strong evidence of a linear relationship between higher consumption along the AHEI-2010, and diminished risks of mortality from all causes and cancer, and the parameter estimates for this index were similar when comparing the two outcomes examined. Moreover, we found evidence of non-linear relationships between adherence to low-carbohydrate indices, in general within the overall and plant-based versions of this index, though the strongest associations were seen in the latter. There were also stronger associations observed for the low-carbohydrate indices when evaluating all-cause mortality rather than cancer mortality. Similar non-linear, albeit weaker, relationships with the aMED index were present, although there was no significant evidence to suggest a protective association with higher intake in a linear fashion. Furthermore, the association of this index with cancer-related mortalities was modified by the tumor site. Finally, a low-carbohydrate index that rewarded greater protein and fat intake from animal-based sources was not significantly associated with the outcomes. Notably, when the relationships with this index were modeled quadratically, it became apparent that it may, potentially, be deleterious with regard to the outcomes.

In this analysis, we implemented the methodology of marginal structural models to account for issues of time-dependent confounding that arise when models involving repeated measures are specified. Indeed, we found that our estimates from unweighted models, in certain specific cases, diverged from those of marginal structural models, suggesting that the unweighted results were confounded at least to some extent. When comparing the results of the marginal structural models to those from the standard unweighted proportional hazards models containing only baseline covariates of dietary intake, it was found that consumption along the same indices produced weaker magnitudes of association in the latter, and these estimates were not statistically significant. Despite this, the most substantial parameter estimates in these models were observed in the AHEI-2010, low-carbohydrate, and plant-based low-carbohydrate indices. Analyses of dietary intake in large cohort studies routinely suffer from a methodological limitation: the failure to account for variation in dietary intake within individuals across time. The present analysis employed an alternative approach to curtail this limitation. Given our results, it is worthy of considering that dietary intake misclassification may have contributed to the null results from standard time-independent Cox models. However, we should also consider and reconcile these non-significant results with a lack of statistical power, especially given that estimates appeared to be trending toward significance and were, generally, in the same direction as those from the MSMs. Nonetheless, specifying a yearly varying metric of dietary intake was a more robust approach for evaluating the role of dietary intake on mortality outcomes.

The biological underpinnings explaining these associations are not fully understood, yet preclinical and *in vitro* analyses highlight potential pathways and players involved. Our study found that the DASH index was not significantly associated with death from all causes nor cancer-specific mortality. This finding lies in contrast to what has previously been observed and reported in other populations. A similar analysis on the NIH-AARP Diet and Health Study found significant inverse associations between baseline aMED and DASH scores, all-cause, and cancer-specific mortalities (57). Similarities between aMED, DASH, and AHEI-2010 and the AHEI-2010 are defined by their emphasis on higher consumption of fruits and vegetables. aMED and AHEI-2010 both reward moderate alcohol consumption, however, differences among them are largely based on how the latter underscores fat quality by rewarding n–3 fatty acid consumption and castigating trans-fat consumption. The fatty acids, eicosapentaenoic acid (EPA) and docosahexaenoic acid (DHA), are shown to be modulators of critical players in several pathways implicated in tumor invasion, angiogenesis, and metastasis (58). Another avenue through which these nutrients may exert effects is through the amelioration of cancer cachexia and by bolstering the efficacy of chemo and radiation therapies. Preclinical and *in vitro* evidence support a role for both EPA and DHA in preventing cachexia-induced muscle loss, regulating aberrant lipogenesis and lipolysis profiles, and prolonging survival (59). We also consider the fact that the AHEI-2010 has a more nuanced computational algorithm than the other indices. Whereas all other indices have discrete scores, the AHEI-2010 provides a range that reflects a more continuous scale. These methodological differences that make the AHEI-2010 stand apart from the other indices may, in part, also explain its superiority as a marker of outcomes in this study and, generally, in chronic disease epidemiology. We must also underscore that the inclusion of alcohol components in the computation of some of these indices may be problematic, specifically within the HNSCC population, despite the strong inverse associations we observed with the AHEI-2010. Previous evidence suggests that continued alcohol intake is deleterious to survival following an HNSCC diagnosis and, thus, we must maintain the results of this study within that framework (12, 18). It is assuring that we still found an inverse association between AHEI-2010, suggesting that other healthful components in the AHEI index may counteract potential negative effects of alcohol on prognosis.

Our results also demonstrated that a low-carbohydrate diet, specifically a low-carbohydrate diet that emphasizes plant-based sources of protein and fat, was inversely associated with all-cause and cancer-specific mortalities within the first 3 years of the study. Interestingly, a low-carbohydrate index that emphasized foods of animal origin was not associated with the outcomes and may have, in contrast, promoted deleterious outcomes though this conclusion could not be explicitly made with the results our analysis generated. Furthermore, given that more robust measures of association were observed in the plant-based iteration of this index, it is conceivable to hypothesize that the significant associations seen in the overall low-carbohydrate index, which were not as strong as in the former, were driven primarily by those scoring highest along with the plant-based version. The Warburg hypothesis is routinely cited in the cancer literature as an approach to ciphering tumor metabolism by abrogating the preferred supply of nutrition, namely glucose and other simple carbohydrates. The conjecture follows that by stifling aerobic glycolysis, tumor progression is theoretically severed (60). Similar to our results, in non-metastatic colorectal carcinoma (CRC), Song et al. reported inverse associations between the same plant-based lower carbohydrate index score and deaths from all-causes and CRC in an analysis of Nurse’s Health Study and Health Professionals Follow-Up Study data (61). It is worth noting that their reported effect sizes were comparable in magnitude to those reported by our team. Nonetheless, evidence from clinical or randomized controlled trials to substantiate this hypothesis is lacking, especially within the HNSCC population. Yet, the results of the present analysis suggest that research endeavors in this capacity may be fruitful.

Other reports in the literature space have described inverse associations between scores on iterations of the AHEI and cancer-specific mortality. This index has broadly been recognized as a predictor of chronic disease incidence and mortality (33). A systematic review and meta-analysis of 13 observational studies found significant inverse relationships between AHEI and all-cause and cancer-specific mortalities (62). Another similar systematic review and meta-analysis found an inverse association between cancer-specific mortality and adherence to AHEI, aMED, and DASH (63). Concerning associations seen in those scoring high along the AHEI-2010, the mechanistic explanations are likely multi-faceted and projected to involve a combination of anti-inflammatory properties, immune-modulating properties, benefits related to increased fiber consumption, the effects of DHA and EPA detailed above, as well as interactions among nutrients that may synergistically confer protection against mortality and cancer progression (64). Thus, the biological sequelae that make these associations plausible are bountiful.

There are implications for clinical best practices considering the results of our study. Nutritional assessment may comprise part of the HNSCC treatment protocol, where patients are screened for malnutrition to evaluate the potential for adverse events during the treatment course (65, 66). Consequently, our results should inform future nutritional interventions in the HNSCC population and highlight the need to monitor patient nutritional protocol through, potentially, the use of FFQs in clinical settings or, at minimum, regular consultation with a Registered Dietitian.

There are several strengths to our analysis. Despite the observational study design and that patients were not randomized to the treatments, repeated measures data in a marginal structural model application produced comparable groups. Observational studies on nutrition suffer from a key limitation: Diet is measured and not randomized. Marginal structural models attempt to abate this issue by creating a pseudo population where treatment (in this analysis, belonging to one of the five quintiles of index scores) is not confounded by the observed history of other measured covariates. Moreover, the results from the sensitivity analyses did not appreciably alter the parameter estimates, and trends across the quintiles (for those indices showing significant results in previous models) were still apparent. There are limitations in the study design worth noting. Though relevant covariates were measured, one of the assumptions underlying marginal structural models is exchangeability or no unmeasured confounding. This assumption was critical to the analysis but cannot be explicitly measured (47). Given the observational nature of this study, residual confounding cannot be ruled out. Another assumption of marginal structural models involves the positivity assumption, which states that exposed and unexposed individuals are found at each level of the confounder variables (47). The violation of this assumption may cause weights to tend to infinity (67). The only categorical level for which this was true was for those individuals with tumors in the hypopharynx, which is due to the remarkably small sample size of this subset (*n* = 10). However, since this violation did not occur in a time-varying covariate, involved a small subset of the study sample, and the ranges of computed weights were reasonable, it is improbable that estimates were biased (47, 67). Nonetheless, participants with tumors in the hypopharynx were collapsed into a single category with tumors of the larynx, given their anatomical proximity and similar profiles in terms of tumor characteristics, to circumvent the issue altogether. Additionally, the data used was not collected with the explicit intent of conducting the present analysis. Time-varying covariates were updated on an annual basis, and there was not an opportunity for finer resolution. Despite this constraint, the FFQ used to measure dietary intake in this study was designed to capture average intake over a given year, and, thus, those data were used accordingly (32). Other sampling-related limitations include the high proportion of white participants, which may diminish the generalizability of these findings to other demographic groups, and the random and systematic biases that arise with the use of food frequency questionnaires. Finally, measurement error and bias in self-reporting dietary data due to symptoms secondary to the disease course and treatment is possible but was not accounted for in this analysis.

We conclude that higher consumption along the AHEI-2010 and a plant-based low-carbohydrate index was inversely associated with all-cause and cancer-specific mortality throughout the first 3 years of follow-up between the aMED index and both outcomes examined, and this relationship was modified by tumor site in the case of cancer-specific mortality. The methodology and thorough approach that generated robust findings were strengths of the analysis, but due to the study’s observational design, future evaluations in the form of randomized controlled trials are needed to substantiate these results.

## Data Availability Statement

The data presented in this study are available on request from the corresponding author. The data are not publicly available due to privacy concerns.

## Ethics Statement

The studies involving human participants were reviewed and approved by the Institutional Review Board of the University of Michigan (Protocol Code: HUM00042189). The patients/participants provided their written informed consent to participate in this study.

## Author Contributions

LR, GW, AM, and AA involved in the conceptualization and design of the study. CM performed the formal analysis and wrote the first draft of the manuscript. CM, KZ, and AA involved in data curation. AA, RS, ZM-E, SR-Z, and AM provided support on methodological and statistical considerations. All authors were involved in the manuscript revision process and approved the final version.

## Conflict of Interest

The authors declare that the research was conducted in the absence of any commercial or financial relationships that could be construed as a potential conflict of interest.

## Publisher’s Note

All claims expressed in this article are solely those of the authors and do not necessarily represent those of their affiliated organizations, or those of the publisher, the editors and the reviewers. Any product that may be evaluated in this article, or claim that may be made by its manufacturer, is not guaranteed or endorsed by the publisher.
