# Supplementary material for: Adherence to a priori-Defined Diet Quality Indices Throughout the Early Disease Course Is Associated With Survival in Head and Neck Cancer Survivors: An Application Involving Marginal Structural Models
Source: Front Nutr. 2022 Apr 25;9:791141. doi: 10.3389/fnut.2022.791141 (PMC9083460; doi:10.3389/fnut.2022.791141)
Supplement: Supplementary file 1 [file Data_Sheet_1.docx]

Supplementary Material

# Supplementary Data

# Supplementary Figures and Tables

| Diet Quality Index | Model^†^ | Q1 | Q2 | Q3 | Q4 | Q5 | *p*_trend_ | *p*_Q5-Q1_ | HR^a^_continuous_ | *p^b^_quadratic_* | |
| --- | --- | --- | --- | --- | --- | --- | --- | --- | --- | --- | --- |
| AHEI-2010 | MSM | 1.00 | 0.22 (0.08-0.61)** | 0.18 (0.07-0.48)** | 0.19 (0.03-1.01) | 0.03 (0-0.14)** | 0.02* | <0.01** | 0.35 (0.20-0.60)** | 0.47 | |
|  | Unweighted | 1.00 | 0.24 (0.09-0.61)** | 0.22 (0.09-0.55)** | 0.25 (0.07-0.85)* | 0.05 (0.01-0.17)** | <0.01** | <0.01** | 0.45 (0.28-0.73)** | 0.41 | |
| aMED | MSM | 1.00 | 0.30 (0.08-1.18) | 0.42 (0.09-1.97) | 0.25 (0.03-1.88) | 0.43 (0.04-4.13) | 0.66 | 0.46 | 0.75 (0.31-1.83) | 0.03* | |
|  | Unweighted | 1.00 | 0.53 (0.17-1.63) | 0.56 (0.16-1.95) | 0.33 (0.09-1.23) | 0.38 (0.09-1.65) | 0.91 | 0.19 | 0.67 (0.37-1.20) | 0.07 | |
| DASH | MSM | 1.00 | 0.51 (0.19-1.32) | 0.76 (0.30-1.96) | 0.52 (0.12-2.20) | 0.68 (0.06-8.03) | 0.72 | 0.76 | 0.69 (0.28-1.68) | 0.25 | |
|  | Unweighted | 1.00 | 0.71 (0.23-2.17) | 1.04 (0.41-2.65) | 0.68 (0.20-2.32) | 0.72 (0.12-4.37) | 0.55 | 0.72 | 0.72 (0.36-1.41) | 0.25 | |
| LC^c^ | MSM | 1.00 | 0.87 (0.43-1.75) | 0.32 (0.13-0.76)* | 0.68 (0.29-1.63) | 0.46 (0.13-1.63) | 0.06 | 0.23 | 0.85 (0.62-1.17) | 0.04* | |
|  | Unweighted | 1.00 | 0.97 (0.50-1.87) | 0.38 (0.18-0.81)* | 0.59 (0.26-1.36) | 0.39 (0.13-1.22) | 0.02* | 0.11 | 0.82 (0.61-1.11) | 0.03* | |
| Animal-Based LC | MSM | 1.00 | 1.02 (0.39-2.66) | 1.41 (0.46-4.36) | 0.70 (0.26-1.87) | 1.12 (0.30-4.19) | 0.25 | 0.86 | 1.01 (0.70-1.44) | 0.04* | |
|  | Unweighted | 1.00 | 0.97 (0.40-2.31) | 1.17 (0.41-3.34) | 0.59 (0.21-1.68) | 0.81 (0.24-2.80) | 0.07 | 0.75 | 0.91 (0.64-1.30) | 0.04* | |
| Plant-Based LC | MSM | 1.00 | 0.35 (0.13-0.96)* | 0.49 (0.17-1.39) | 0.40 (0.14-1.16) | 0.34 (0.09-1.34) | 0.54 | 0.12 | 0.73 (0.53-1.03) | 0.03* | |
|  | Unweighted | 1.00 | 0.69 (0.23-2.12) | 0.77 (0.28-2.11) | 0.62 (0.23-1.65) | 0.56 (0.19-1.71) | 0.73 | 0.31 | 0.82 (0.62-1.09) | 0.03* | |
| **Supplementary Table 1.** Hazard ratios^†^ and 95% confidence intervals for all-cause mortality from both marginal structural models and unweighted models across each dietary index from the sensitivity analysis where subjects were censored at their second visit if they had missing dietary data but were otherwise followed up longitudinally. This analysis used 934 person-years of available data and censored all individuals with missing follow-up dietary data at the end of the first year of observation.  **  *p* < 0.01  * *p* < 0.05  ^†^ All models adjusted for baseline age, sex, tumor site, stage, HPV status, treatment modality, ACE-27, highest level of education attained, index score measured at the previous encounter, smoking status, caloric intake, and BMI.  ^a^ Hazard ratio (HR) corresponding to a standard deviation increase in the diet index score  ^b^ Wald test *p*-value for a quadratic polynomial term  ^c^ Low Carbohydrate (LC) | | | | | | | | | | |  |

| Diet Quality Index | Model^†^ | Q1 | Q2 | Q3 | Q4 | Q5 | *p*_trend_ | *p*_Q5-Q1_ | | HR^a^_continuous_ | | *p^b^_quadratic_* | |
| --- | --- | --- | --- | --- | --- | --- | --- | --- | --- | --- | --- | --- | --- |
| AHEI-2010 | MSM | 1.00 | 0.26 (0.05-1.29) | 0.40 (0.07-2.35) | 0.65 (0.12-3.55) | 0.12 (0.02-0.92)* | 0.02* | 0.04* | | 0.57 (0.33-0.98)* | | 0.47 | |
|  | Unweighted | 1.00 | 0.24 (0.06-0.88)* | 0.25 (0.07-0.92)* | 0.58 (0.16-2.12) | 0.09 (0.02-0.42)** | <0.01** | <0.01** | | 0.57 (0.37-0.88)* | | 0.45 | |
| aMED | MSM | 1.00 | 0.69 (0.16-2.98) | 0.82 (0.12-5.57) | 0.72 (0.09-5.94) | 1.10 (0.09-12.75) | 0.30 | 0.94 | | 1.09 (0.42-2.82) | | 0.04* | |
|  | Unweighted | 1.00 | 0.80 (0.28-2.25) | 0.58 (0.14-2.47) | 0.40 (0.09-1.74) | 0.41 (0.08-2.09) | 0.82 | 0.28 | | 0.71 (0.36-1.41) | | 0.08 | |
| DASH | MSM | 1.00 | 2.34 (0.75-7.35) | 1.95 (0.42-9.13) | 2.77 (0.22-34.56) | 4.66 (0.21-103.42) | 0.91 | 0.33 | | 1.21 (0.34-4.31) | | 0.20 | |
|  | Unweighted | 1.00 | 3.04 (0.94-9.85) | 2.21 (0.62-7.86) | 2.32 (0.34-15.81) | 2.26 (0.18-28.52) | 0.63 | 0.53 | | 0.98 (0.40-2.41) | | 0.32 | |
| LC^c^ | MSM | 1.00 | 0.68 (0.27-1.66) | 0.27 (0.09-0.77)* | 0.54 (0.19-1.53) | 0.39 (0.10-1.54) | 0.08 | 0.18 | | 0.72 (0.50-1.05) | | 0.52 | |
|  | Unweighted | 1.00 | 0.60 (0.23-1.54) | 0.24 (0.09-0.63)** | 0.47 (0.17-1.27) | 0.33 (0.09-1.19) | 0.06 | 0.09 | | 0.69 (0.49-0.97)* | | 0.39 | |
| Animal-Based LC | MSM | 1.00 | 1.16 (0.38-3.54) | 0.83 (0.30-2.25) | 0.26 (0.07-0.96)* | 0.64 (0.16-2.61) | 0.26 | 0.53 | | 0.79 (0.49-1.28) | | 0.99 | |
|  | Unweighted | 1.00 | 1.02 (0.37-2.80) | 0.58 (0.19-1.71) | 0.20 (0.05-0.75)* | 0.51 (0.14-1.88) | 0.18 | 0.31 | | 0.74 (0.48-1.15) | | 0.91 | |
| Plant-Based LC | MSM | 1.00 | 0.40 (0.13-1.26) | 0.55 (0.18-1.67) | 0.39 (0.12-1.26) | 0.27 (0.05-1.39) | 0.14 | 0.12 | | 0.63 (0.45-0.90)* | | 0.02* | |
|  | Unweighted | 1.00 | 0.52 (0.18-1.49) | 0.72 (0.27-1.93) | 0.52 (0.19-1.43) | 0.39 (0.11-1.37) | 0.19 | 0.14 | | 0.70 (0.53-0.92)* | | 0.02* | |
| **Supplementary Table 2.** Hazard ratios^†^ and 95% confidence intervals for cancer-specific mortality from both marginal structural models and unweighted models across each dietary index from the sensitivity analysis where subjects were right-censored at their second visit if they had missing dietary data but were otherwise followed up longitudinally. This analysis used 934 person-years of available data and censored all individuals with missing follow-up dietary data at the end of the first year of observation.  **  *p* < 0.01  * *p* < 0.05  ^†^ All models adjusted for baseline age, sex, tumor site, stage, HPV status, treatment modality, ACE-27, highest level of education attained, index score measured at the previous encounter, smoking status, caloric intake, and BMI.  ^a^ Hazard ratio (HR) corresponding to a standard deviation increase in the diet index score  ^b^ Wald test *p*-value for a quadratic polynomial term  ^c^ Low Carbohydrate (LC) | | | | | | | | |  | |  | |  |

| *All-Cause Mortality* | | | *Cancer-Specific Mortality* | | |
| --- | --- | --- | --- | --- | --- |
| Diet Quality Index | Effect Modifier | *p_LRT_* | Diet Quality Index | Effect Modifier | *p_LRT_* |
| AHEI-2010 | Cancer Stage | 0.36 | AHEI-2010 | Cancer Stage | 0.06 |
| AHEI-2010 | HPV Status | 0.73 | AHEI-2010 | HPV Status | 0.69 |
| AHEI-2010 | Tumor Site | 0.86 | AHEI-2010 | Tumor Site | 0.64 |
| aMED | Cancer Stage | 0.54 | aMED | Cancer Stage | 0.32 |
| aMED | HPV Status | 0.69 | aMED | HPV Status | 0.48 |
| aMED | Tumor Site | 0.08 | aMED | Tumor Site | 0.04* |
| DASH | Cancer Stage | 0.07 | DASH | Cancer Stage | 0.11 |
| DASH | HPV Status | 0.82 | DASH | HPV Status | 0.80 |
| DASH | Tumor Site | 0.73 | DASH | Tumor Site | 0.12 |
| Low Carbohydrate | Cancer Stage | 0.10 | Low Carbohydrate | Cancer Stage | 0.38 |
| Low Carbohydrate | HPV Status | 0.30 | Low Carbohydrate | HPV Status | 0.51 |
| Low Carbohydrate | Tumor Site | 0.97 | Low Carbohydrate | Tumor Site | 0.29 |
| Animal-Based Low Carbohydrate | Cancer Stage | 0.06 | Animal-Based Low Carbohydrate | Cancer Stage | 0.10 |
| Animal-Based Low Carbohydrate | HPV Status | 0.50 | Animal-Based Low Carbohydrate | HPV Status | 0.59 |
| Animal-Based Low Carbohydrate | Tumor Site | 0.72 | Animal-Based Low Carbohydrate | Tumor Site | 0.28 |
| Plant-Based Low Carbohydrate | Cancer Stage | 0.22 | Plant-Based Low Carbohydrate | Cancer Stage | 0.72 |
| Plant-Based Low Carbohydrate | HPV Status | 0.58 | Plant-Based Low Carbohydrate | HPV Status | 0.28 |
| Plant-Based Low Carbohydrate | Tumor Site | 0.99 | Plant-Based Low Carbohydrate | Tumor Site | 0.53 |
| **Supplementary Table 3.** Likelihood ratio test *p*-values comparing MSMs with and without interaction terms between variables listed in the Effect Modifier column and the scaled numerical variable of the diet quality index indicated.  **  *p* < 0.01  * *p* < 0.05  All models adjusted for baseline age, sex, tumor site, stage, HPV status, treatment modality, ACE-27, highest level of education attained, index score measured at the previous encounter, smoking status, caloric intake, and BMI. | | | | | |

| Model | Diet Index | Site | HR^a^_continuous_ |
| --- | --- | --- | --- |
| MSM | aMED | Larynx/Hypopharynx | 1.78 (0.58-5.46) |
| MSM | aMED | Oral Cavity | 0.99 (0.31-1.02) |
| MSM | aMED | Oropharynx | 0.73 (0.21-0.80)** |
| **Supplementary Table 4.** Stratified HR estimates across levels of tumor site.  **  *p* < 0.01  * *p* < 0.05  ^†^ All models adjusted for baseline age, sex, tumor site, stage, HPV status, treatment modality, ACE-27, highest level of education attained, index score measured at the previous encounter, smoking status, caloric intake, and BMI.  ^a^ Hazard ratio (HR) corresponding to a standard deviation increase in the diet index score. Modified HRs were estimated by including an interaction term with the diet quality index variable in the model. | | | |

## Supplementary Figures


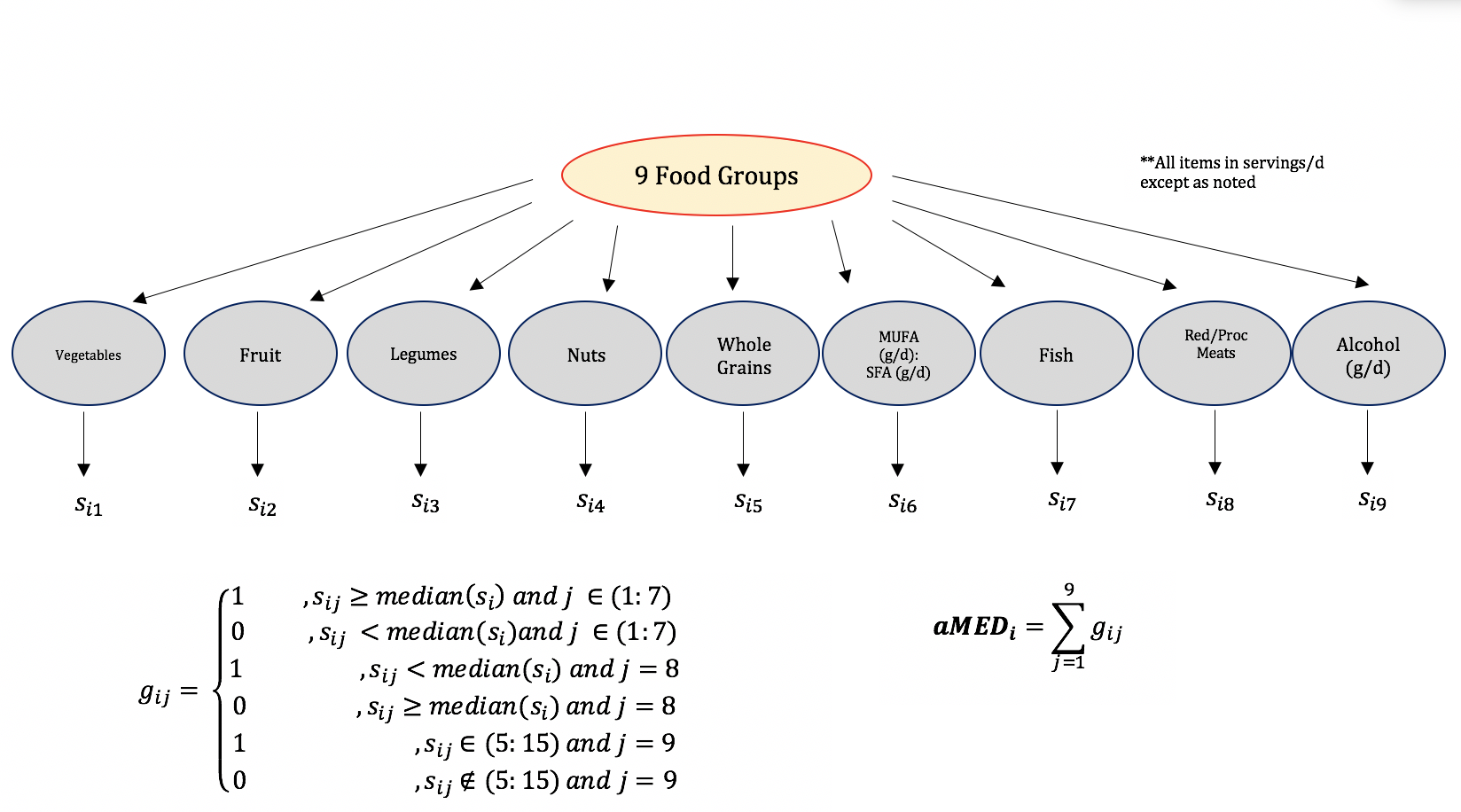


**Supplementary Figure 1.** Scoring algorithm for the aMED diet quality index.


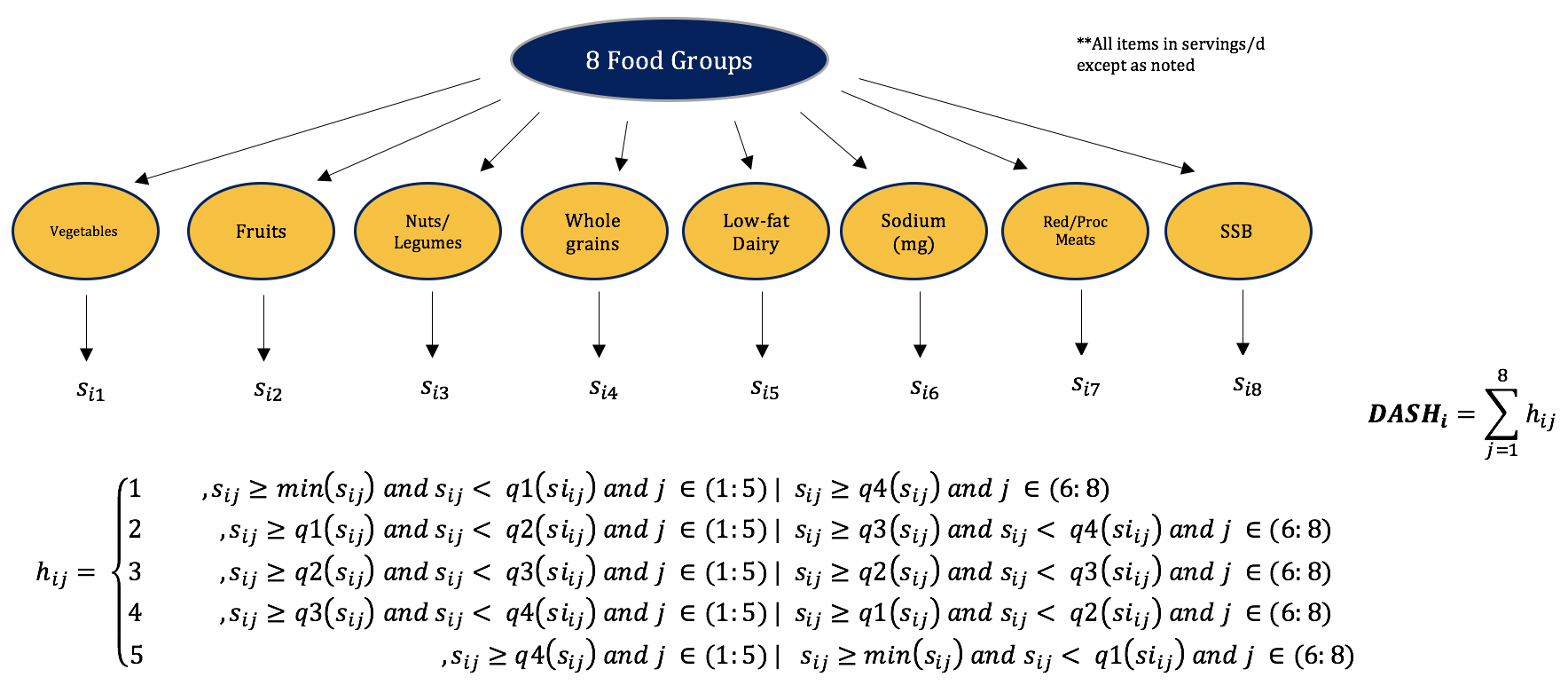


**Supplementary Figure 2.** Scoring algorithm for the DASH diet quality index.


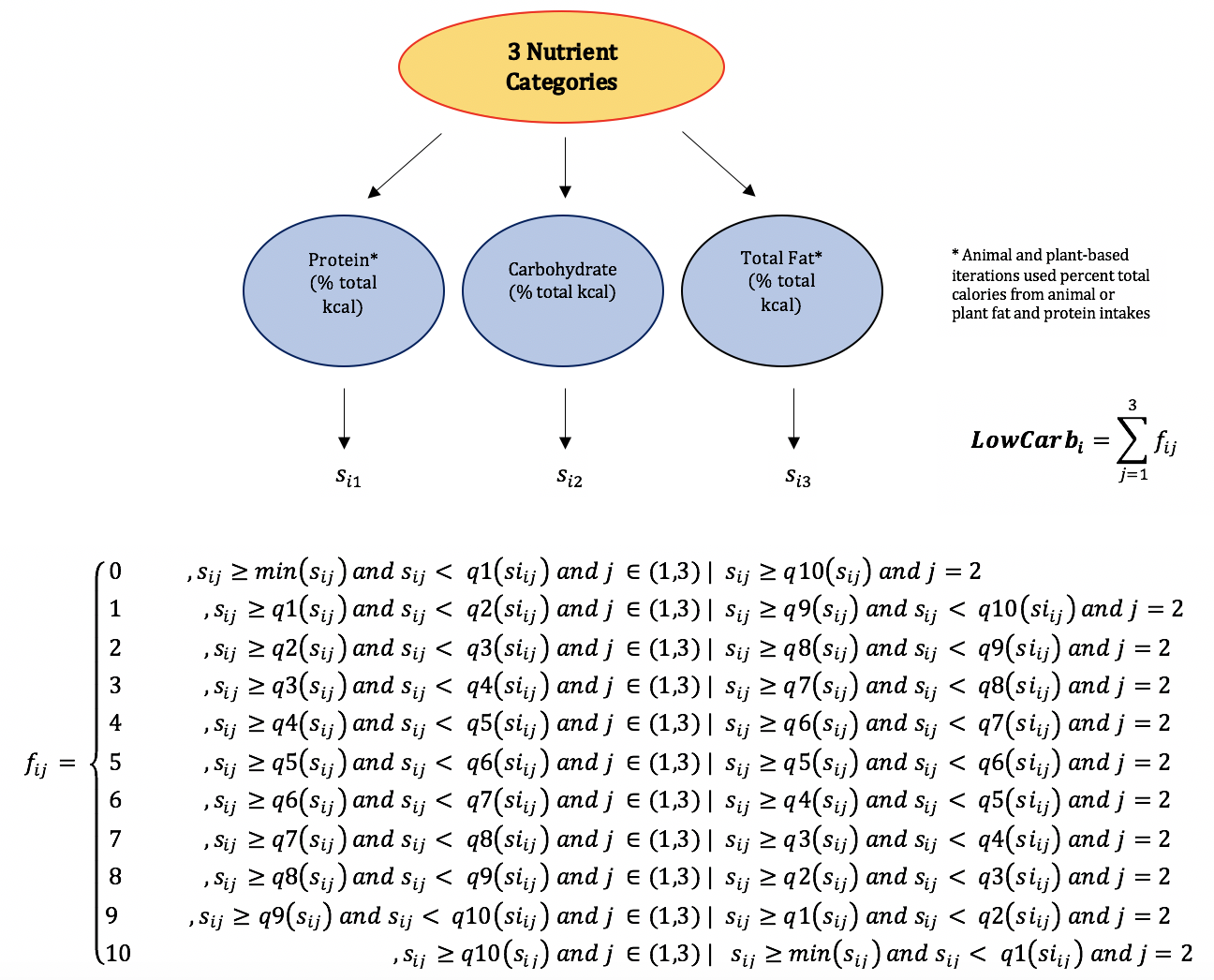


**Supplementary Figure 3.** Scoring algorithms for the low-carbohydrate diet quality indices.
